# J-Integral Experimental Reduction Reveals Fracture Toughness Improvements in Thin-Ply Carbon Fiber Laminates with Aligned Carbon Nanotube Interlaminar Reinforcement

**DOI:** 10.1021/acsami.3c17333

**Published:** 2024-04-16

**Authors:** Carolina Furtado, Reed Kopp, Xinchen Ni, Carlos Sarrado, Estelle Kalfon-Cohen, Brian L. Wardle, Pedro P. Camanho

**Affiliations:** † DEMec, Faculdade de Engenharia, Universidade do Porto, Rua Dr. Roberto Frias, s/n, Porto 4200-465, Portugal; ‡ INEGI, Instituto de Ciência e Inovação em Engenharia Mecânica e Engenharia Industrial, Rua Dr. Roberto Frias, 400, Porto 4200-465, Portugal; § Department of Aeronautics and Astronautics, Massachusetts Institute of Technology, 77 Massachusetts Avenue, Cambridge, Massachusetts 02139, United States; ∥ Department of Mechanical Engineering, Massachusetts Institute of Technology, 77 Massachusetts Avenue, Cambridge, Massachusetts 02139, United States; ⊥ AMADE, Polytechnic School, Universitat de Girona, Campus Montilivi s/n, Girona 17073, Spain; # AMTEC Composites, C/Pic de Peguera 15, Girona 17003, Spain

**Keywords:** A. polymer-matrix composites (PMCs), B. mechanical testing, C. carbon nanotubes, D. fracture toughness, E. delamination

## Abstract

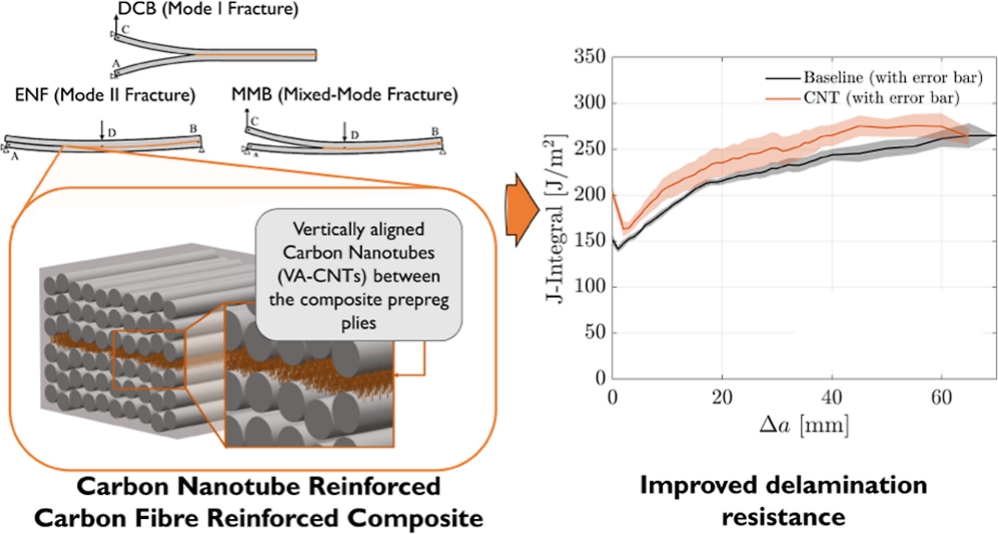

The Mode I, Mode II, and mixed-mode interlaminar failure behavior of a thin-ply (54 gsm) carbon fiber-epoxy laminated composite reinforced by 20 μm tall *z*-direction-aligned carbon nanotubes (CNTs), comprising ∼50 billion CNT fibers per cm^2^, is analyzed following J-integral-based data reduction methods. The inclusion of aligned CNTs in the ply interfaces provides enhanced crack resistance, resulting in sustained crack deflection from the reinforced interlaminar region to the intralaminar region of the adjacent plies, i.e., the CNTs drive the crack from the interlaminar region into the plies. The CNTs do not appreciably increase the interlaminar thickness or laminate weight and preserve the intralaminar microfiber morphology. Improvements of 34 and 62% on the Mode I and Mode II initiation fracture toughness, respectively, are observed. This type of interlaminar nanoreinforcement effectively drives crack propagation from the interface to within the ply where the crack propagates parallel to the interlaminar region, providing new insight into previously reported strength and fatigue performance increases. These findings extend to industries where lightweight and durable materials are critical for improving the structural efficiency.

## 1 Introduction

**Figure 1:**
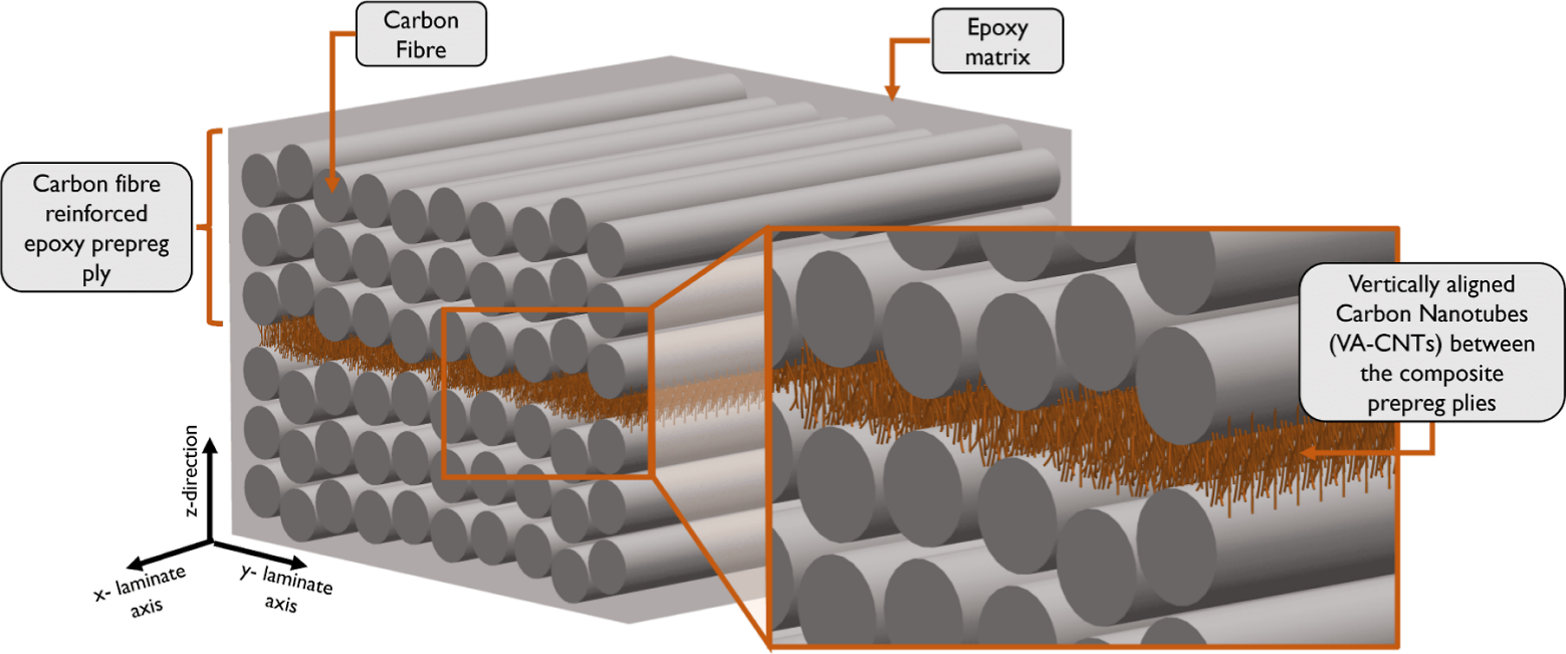
Schematic representation of nanostitch where VA-CNTs are introduced between the plies (two 0° deg plies in the illustration) of a prepreg-based laminate, resulting in a nanoengineered multiscale architecture.

Interest has been growing in the development of nanostructured hybrid (multiscale) composite materials, where nanoparticles such as carbon nanotubes (CNTs) are used alongside microscale-fiber composite laminates.^1^ CNTs introduce additional energy dissipating mechanisms during crack propagation that can result in significant improvement of the matrix-dominated properties of traditional composites and consequently improve their mechanical behavior. Vertically aligned CNTs (VA-CNTs), where the individual CNTs grow aligned perpendicular to the substrate, in forests with an areal density of ∼1 vol % and inter-CNT spacing of ∼80 nm provide an opportunity for engineering properties due to the aligned nature of the nanofibers,^2^ similar to aligned-microfiber traditional advanced composites, leading to a textured nature that can provide engineering advantage in various properties.^3−5^ Recently, a reinforcing technique that consists of introducing vertically aligned CNTs (VA-CNTs) between the plies of a prepreg-based laminate, resulting in a nanoengineered architecture, has been developed and termed “nanostitching”.^6^ This technology, schematically shown in Figure 1, guarantees a good dispersion of generally aligned and continuous CNTs across the interlaminar region, resulting in improvements in the interlaminar shear strength^7^ and fracture toughness,^6^ a significant increase in fatigue life, particularly under low-stress regimes,^7^ and delay/suppression of interlaminar subcritical damage and consequent increase in the design strengths (damage onset and ultimate strength) in composite subcomponents^8^ (as opposed to other through-thickness reinforcement types that rely on microfiber bridging as the main toughening mechanism such as stitching, z-pinning, and weaving^9−15^). These results are summarized in Table 1. Furthermore, the improved thermal^16−21^ and electrical^20−22^ properties of VA-CNTs can be leveraged to reduce manufacturing costs and increase composite multifunctionality.^3,23−27^

**Table 1:** Summary of the Outcomes of the Past Structural Test Results on Nanostitched Laminates^a^

mechanical test	study	Mat. type	outcome (vs baseline)
short-beam shear (SBS)^7^	Exp.	standard	+9% in static strength, +2–3X fatigue life
short-beam shear (SBS)^28^	Exp.	thin-ply	+10% from thin-plies and +5% from CNTs
double edge-notched tension^29^	Exp.	standard	+8% in notched strength
critical bolt-bearing^8^	Exp.	standard	+30% in critical bearing strength
open-hole compression^8^	Exp.	standard	+14% in strength
L-shape bending energy^8^	Exp.	standard	+31% in energy to break

a All tests were performed on standard-thickness composites (0.125 mm) except the study performed by Kalfon-Cohen^28^ (0.054 mm).

More recently, Kalfon-Cohen et al.^28^ attempted for the first time to combine thin-ply laminates with nanoreinforced interfaces to investigate the potential improvements in the interlaminar strength and toughness that could be obtained by reinforcing thin-ply laminates with CNTs as compared to baseline conventional-grade laminates. A synergistic strength effect of nanostitching and thin-ply lamination was found: the use of thin-ply increased the interlaminar shear strength by 10% over standard-grade plies (69.9 ± 2.4 to 76.9 ± 1.1 MPa), and the selective reinforcement of the interfaces further increased it by 5% (76.9 ± 1.1 to 80.6 ± 0.6 MPa). Moreover, a complete suppression of delamination planes in the nanoreinforced interfaces was found, which confirms the effectiveness of the nanostitch reinforcement technique. A conservative 15% increase of the Mode I and Mode II fracture toughnesses of the nanoreinforced interfaces was numerically estimated based on the finite element analysis of the short-beam shear (SBS) samples. However, proper experimental characterization of the fracture toughness of these reinforced interfaces was never performed. To characterize the interlaminar crack propagation in nanoreinforced thin-ply carbon fiber-epoxy systems, the Mode I, Mode II, and mixed-mode crack resistance curves associated with crack propagation in nanostitched thin-ply interfaces are experimentally determined here for the first time, shedding new light on the failure mechanisms of nanostitched interfaces. While widely adopted ASTM standards^30−32^ for assessing fracture toughness in fiber-reinforced polymer composites are based on linear elastic fracture mechanics (LEFM), thus disregarding nonlinear deformation at the crack front and bridging mechanisms, our approach differs: we used J-integral closed-form solutions.^33−35^ The J-integral,^36^ developed by James Rice, is a nonlinear fracture mechanics concept used to quantify the energy release rate near the tip of a crack in a material, which provides a measure of not only the elastic deformation energy but also the plastic deformation energy and bridging effects associated with the propagation of a crack, offering insight into the resistance to fracture of toughened materials. When applied to measuring the interface fracture toughness of the composite material, it requires the experimental determination of the load and sample arm rotation angles and offers two advantages against the standard LEFM-based approaches: (i) it does not rely on the experimental measurement of the crack length, significantly reducing uncertainties in fracture toughness measurements, and (ii) it can account for larger fracture process zones and additional toughening effects, resulting, for example, from nanoreinforcement to thermoplastic-particle reinforcement of elevated temperature testing. Here, both the ASTM-standard-proposed and J-integral data reduction approaches are used and compared. For the baseline specimens that have only an interlaminar fracture, the ASTM and J-integral approaches agree. However, the nanostitched interfaces are too tough to allow crack propagation, and the crack bifurcates into the intralaminar region and then propagates there in a plane parallel to the interlaminar region, highlighting the limitations of the LEFM-based characterization methods in accurately determining fracture toughness for these materials, which may require J-integral-based solutions for accurate characterization.

## 2 Test Setup and Data Reduction Methods

The ASTM and ISO standards^30−32,37,38^ proposed to measure Mode I, Mode II, and mixed-mode interlaminar fracture toughness are based on LEFM, where the main assumption is that the nonlinear deformation at the crack front is small in comparison to any of the specimen’s dimensions and crack length. The method has been applied to fiber-reinforced polymer composites for decades; however, the determination of the fracture toughness and crack resistance curves using these methods relies on the accurate measurement of crack length, which can be particularly difficult for Mode II and mixed-mode tests, or on an indirect determination of an “equivalent” crack length. However, the main hypothesis that the nonlinear deformation at the crack front and the bridging mechanisms that may be present along the wake of the crack have a negligible effect might not apply for all material systems and testing conditions, including adhesive joints^39^ or reinforced interfaces (e.g., nanoreinforced and thermoplastic-particle-reinforced) or elevated-temperature testing,^40^ as the crack length itself is not well-defined. For significant engineered interlaminar interfaces, LEFM assumptions do not hold, and more advanced methods, such as the J-integral, are required.

J-integral closed-form solutions for interlaminar fracture tests have been proposed for Mode I,^36,41−43^ Mode II,^34,44−46^ and mixed-mode,^35,47−49^ but the ASTM and ISO standard methods are still usually preferred over these solutions as they have been well-established in industry and academia for many decades. In this work, the J-integral-based solutions proposed by Paris and Paris,^33^ Stigh et al.,^34^ and Sarrado et al.^35^ are used to characterize the interlaminar fracture toughness of thin-ply nanoreinforced interfaces. These J-integral closed-form solutions rely on the rotation angle of the load introduction points instead of the crack length, reducing the uncertainty of the toughness and greatly simplifying the data postprocessing.

The Mode I, Mode II, and mixed-mode specimens were tested on an MTS Insight equipped with a 10 kN load cell under displacement control at speeds of 5, 1, and 0.5 mm/min, respectively. Capacitive dielectric-liquid-based inclinometers (model NA3-30 by SEIKA Mikrosystemtechnik GmbH) were used to measure the rotational angles at the load introduction points. Despite not being required to calculate the J-integral, the crack length was monitored from the photos of the cross-section of the specimens recorded with a Canon EOS 550D camera so that the crack resistance curves (J-integral as a function of the crack length) could be obtained and the toughness could also be reduced from test data based on the ASTM standards for comparison. The load, displacement, and rotation angles were recorded at a frequency of 20 Hz, and the crack length was monitored from the photos of the cross-section of the specimens and recorded every 0.5 s. A schematic representation of the experimental setup and instrumentation is shown in Supporting Information A.

The specimen preparation is presented in Section 2.1, and the particular data reduction methods and instrumentation for each type of test are explained in Section 2.2.

### 2.1 Specimen Preparation

20 ±5 μm-tall VA-CNT forests were grown in a 2″ tube furnace (Lindberg/Blue M) by chemical vapor deposition on 30 mm × 120 mm silicon wafer substrates.^8^ The VA-CNT forests, composed of 8 nm diameter multiwall CNTs (2–3 walls), have an areal density of ∼1 vol % and inter-CNT spacing of ∼80 nm, corresponding to ∼10^9^ to 10^10^ multiwalled CNTs per cm^2^.^50−53^ The final stage of the process included a water-assisted delamination step that allowed the CNT forests to be easily removed from the silicon wafers.

Aerospace-grade 54 gsm thin-ply HTS40/Q-1112 carbon fiber-epoxy prepreg supplied by Teijin Carbon America (formerly Toho Tenax) was utilized.^28^ Baseline (B) and nanoreinforced (CNT) unidirectional [0_60_] laminates were manufactured. The middle two plies were laid up with a mismatch angle of ±2°, and a 60 mm long and 0.025 mm thick PTFE insert was introduced in the midplane of each plate to create the precrack. The nanostitched samples were manufactured, including the 30 mm × 120 mm VA-CNT array in the middle interface, as described in ref (7) and shown in Supporting Information B: first, the silicon wafer with VA-CNTs was inverted onto the prepreg, pressure was applied to the backside of the wafer, and finally, the wafer was removed, leaving the CNTs in the prepreg. The setup was then continued, resulting in CNT-rich ply interfaces. The panels were cured in an autoclave according to the manufacturer specifications: 30 min at 80 °C at 0 bar, followed by 90 min at 130 °C at 7 bar(g). Previous work on the same CFRP material system and VA-CNT forest height^28,54^ shows by means of a scanning electron microscope and micrographs that, following this method, the CNTs are effectively transferred onto the prepreg, properly filling the interlaminar resin-rich area and effectively bridging the two plies.

Five specimens per laminate configuration (B and CNT) and test configuration (double cantilever beam or DCB, end-notch flexure or ENF, and mixed-mode bending or MMB) were cut from the plates by using a diamond-coated circular wet saw. Before testing, one of the surfaces of the specimens was roughened using sandpaper, cleaned with acetone, painted white, and marked at every millimeter so that the crack length could be monitored. Table 2 summarizes the manufactured specimens.

**Table 2:** Description of the Stacking Sequence, CNT Position, and Dimensions of the Specimens

	baseline			CNT		
	DCB	ENF	MMB	DCB	ENF	MMB
layup	[0_60_]	[0_60_]	[0_60_]	[0_60_]	[0_60_]	[0_60_]
CNT position				middle interface	middle interface	middle interface
number of specimens	5	5	5	5	5	5
thickness (mm)	3.46 ± 0.05	3.64 ± 0.01	3.58 ± 0.03	3.36 ± 0.05	3.66 ± 0.03	3.49 ± 0.05
width (mm)	25.13 ± 0.02	25.09 ± 0.02	25.16 ± 0.02	25.05 ± 0.02	25.08 ± 0.02	25.12 ± 0.01
length (mm)	250 ± 1	250 ± 1	250 ± 1	250 ± 1	250 ± 1	250 ± 1

### 2.2 Data Reduction Methods

A representation of the test setups of the DCB, ENF, and MMB specimens is shown in Supporting Information A.

The J-integral for Mode I was calculated as proposed by Paris and Paris^33^
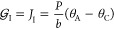
1where *P* is the applied load, *b* is the width of the specimen, and θ_*i*_ are the angles at the load introduction points (see Supporting Information A), defined as positive counterclockwise. Crack advance was monitored to both compute the ASTM-standard  and plot the ASTM-standard and J-integral-derived crack resistance curves.

The determination of the Mode II crack resistance curves requires the crack propagation to be stable, which occurs for an initial crack length-to-half span length (*a*_0_/*L*) ratio higher than 0.7^a^. Since a half span of 60 mm was used here (rather than the 50 mm suggested by the ASTM standard^31^) to increase the crack propagation length, an initial crack length of 42 mm was selected to ensure stable crack propagation during the test.

The J-integral for Mode II is calculated as proposed by Stigh et al.^34^
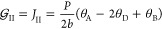
2

For mixed Mode I–Mode II tests, the distance between supports was set to 150 mm to achieve a (stable) crack propagation length that is longer than that proposed by the standard, and the initial crack length was set to 45 mm. The MMB lever arm was determined following the ASTM standard^32^ for a targeted mixed-mode ratio of 50%.

The J-integral for mixed-mode bending was calculated as proposed by Sarrado et al.^35^
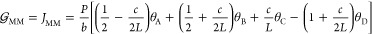
3

## 3 Experimental Test Results

The Mode I, Mode II, and mixed-mode test results are presented in Sections 3.1–3.3 using the J-integral data reduction approach and compared in Section 4. A common theme in all specimens is that the CNT reinforcement causes the interlaminar crack to bifurcate into and remain propagating in the intralaminar region as a crack parallel to the interlaminar plane.

### 3.1 Mode I Test Results

**Figure 2:**
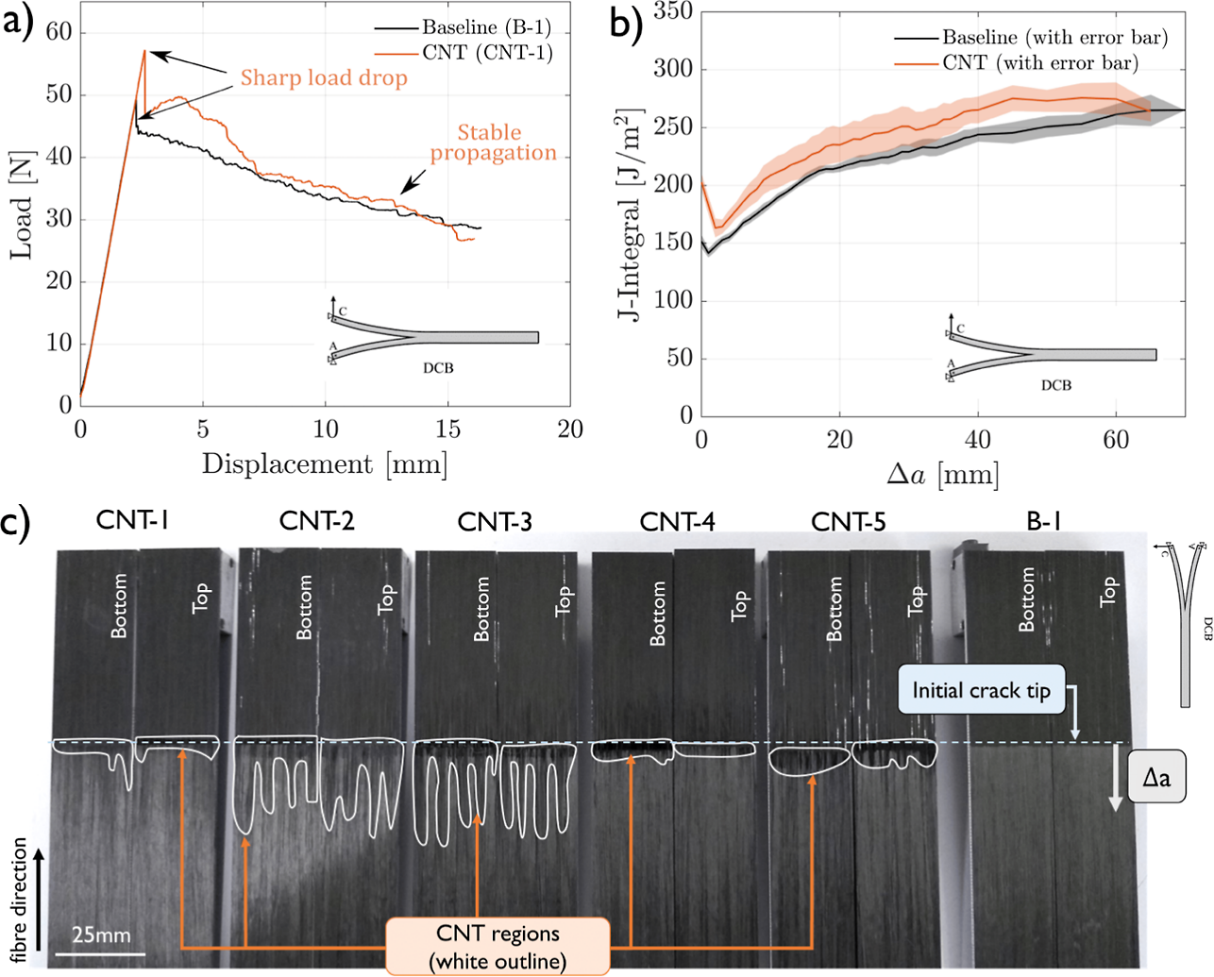
Mode I test results: (a) representative Mode I load–displacement curves, (b) average Mode I crack resistance curves of baseline (B) and CNT-reinforced (CNT) samples determined following eq 1, and (c) top and bottom crack surfaces of one baseline sample (B-1) and all CNT Mode I samples. The CNT-rich crack surfaces are highlighted with a white contour.

All of the specimens showed a linear elastic behavior up to the point of maximum load, at which point the load dropped sharply, as shown for representative specimens in Figure 2a. The specimens were not precracked, and consequently, the cracks started propagating from the area at the edge of the release film. The vicinity of the release film is typically an uncontrolled (and uncharacterized) resin-rich area, and the corresponding crack is likely not as sharp as the crack tip resulting from propagation. The first load drop observed after the peak load in every test is attributed to the initial crack geometry and resin content. Even though the point of maximum load might yield unrealistically high values of the fracture toughness, it was used to calculate the J-integral initiation toughness so that a comparison to the broader literature and between the two material configurations could be made. From the load and rotation angles at the load introduction points obtained for each specimen, the Mode I J-integral was determined following eq 1 and plotted as a function of the crack length. The average -curves for both configurations are shown in Figure 2b, including the dispersion, represented by the standard error based on the 5 samples tested per configuration.

**Figure 3:**
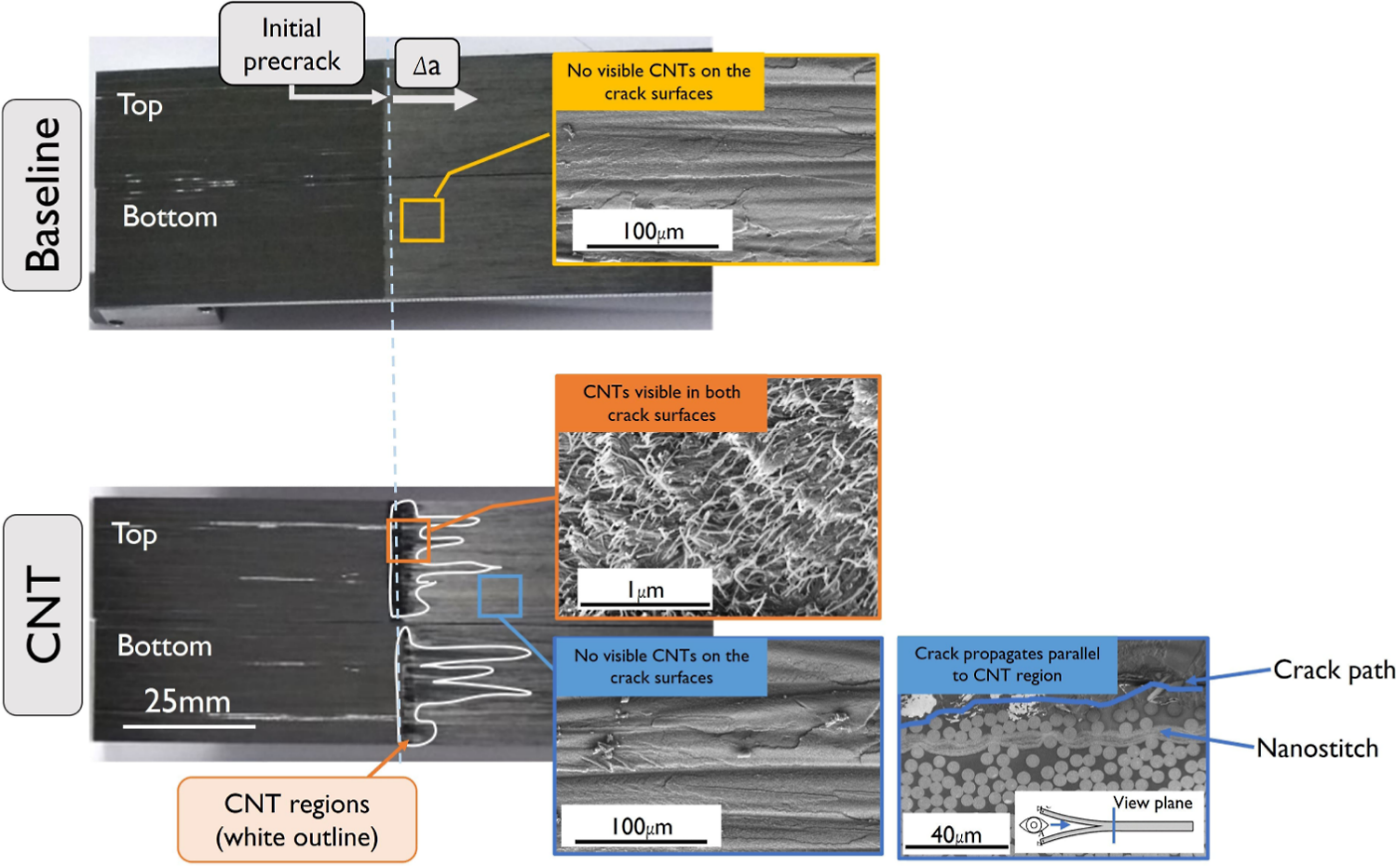
SEM analysis of the crack surfaces of a glossy dark region in a baseline specimen (in yellow), a dark matte region corresponding to a CNT-rich crack surface (in orange), and a glossy dark region in a nanostitched specimen corresponding to intralaminar crack propagation (in blue).

In addition to the -curve reduction, it is important to relate the crack resistance curves to the crack path. To analyze the crack path, the top and bottom crack surfaces of the baseline and CNT-reinforced fractured samples were analyzed postmortem. From optical and scanning electron microscopy (SEM) analysis on fractured Mode I samples shown in Figure 3, it was concluded that dark matte areas (highlighted with a white contour) correspond to CNT-rich regions where the CNTs can be observed on both surfaces (in orange). If a dark matte area is visible on the top and bottom crack surfaces, then the crack is interpreted to have propagated through the CNT reinforcement. Lighter glossy areas correspond to resin-/(micro) fiber-rich areas where CNTs are not present at the crack surface. This is the type of crack surface typically observed for baseline samples since no CNTs are present (in yellow). However, they are also visible in CNT-reinforced samples, which suggests that, during propagation, the crack can deviate from the CNT-reinforced region into a resin-fiber-rich area (in blue).

Through the analysis of the crack surfaces of all the broken CNT-reinforced Mode I samples (Figure 2c), it was consistently observed that the area near the release film is a CNT-rich area, but as the crack progresses, the CNT-rich area decreases significantly, vanishing within 5–25 mm of Δ*a*, depending on the sample. The crack length at which bifurcation occurs is unstudied and likely influenced by details of the crack-starter film (and region from which crack initiates), the local microfiber morphology, and other factors such as manufacturing artifacts^55^ and, of course, any uncontrolled manufacturing defects. The bifurcated crack path is an interesting topic for future work. The results demonstrate that, similar to the findings reported by Ni et al.^56^ for standard-thickness epoxy CFRP prepreg nanoreinforced specimens, near the release film, the crack propagated through the reinforced area and then progressively deviated from this area into the unreinforced intralaminar region, i.e., the inclusion of VA-CNTs provides additional resistance, attributed to additional dissipative mechanisms, such as nanotube breakage, pull-out, sword-in-sheath, and debonding,^16^ resulting in crack bifurcation and sustained crack deflection from the reinforced interlaminar region to the intralaminar region of the plies. Similar to ref (56), we find that the Mode I crack propagates in the intralaminar region approximately 1–2 (micro)fiber diameters into one of the adjacent plies, parallel to the interlaminar plane, as a “Mode I intralaminar crack”.

The values of the fracture toughness were calculated for Δ*a* = 0 mm at the point of maximum load since the cracks only propagate through the CNT-reinforced region for very small crack lengths. A 34% increase in the interlaminar Mode I initiation value of the fracture toughness is measured due to the nanoreinforcement. For Δ*a* > 0 mm, the fracture toughness values measured and presented in the crack resistance curves are not directly related to the CNT-reinforced interface but to a combination of reinforced interlaminar and unreinforced intralaminar regions in the early stage (Δ*a* < 5–25 mm) of crack deflection where the -curve is rising, but thereafter measure the toughness of the intralaminar regions where the crack propagates parallel to the interlaminar region but inside one of the adjacent plies.

### 3.2 Mode II Test Results

**Figure 4:**
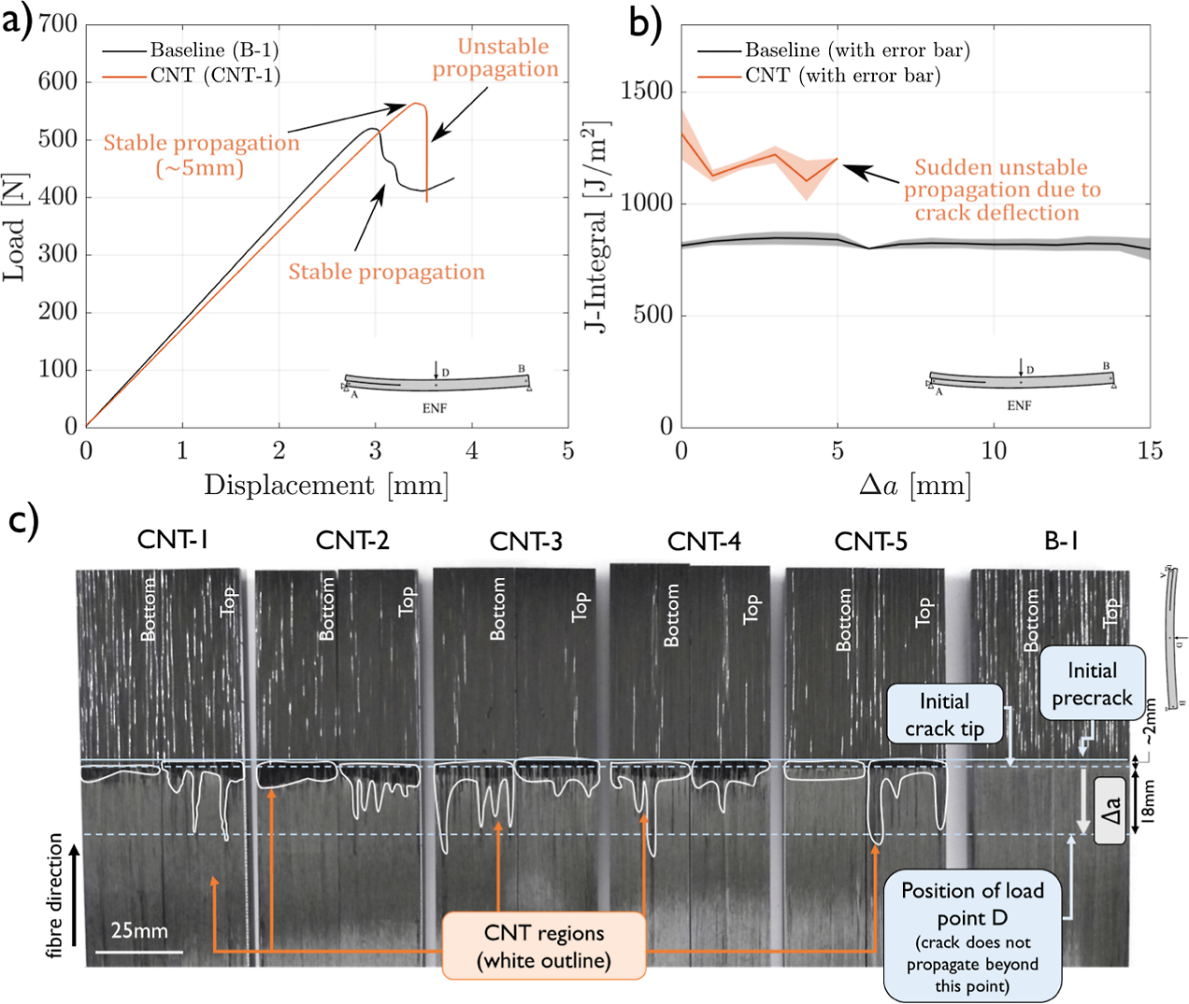
Mode II test results: (a) representative Mode II load–displacement curves, (b) average Mode II crack resistance curves of baseline (B) and CNT-reinforced (CNT) samples determined following eq 2, and (c) top and bottom crack surfaces of one baseline sample (B-1) and all CNT Mode II samples. The CNT-rich crack surfaces are highlighted with a white contour.

Despite the modification of the test setup, where the *a*_0_/*L* ratio was selected to guarantee stable crack propagation (see Section 2.2), initial tests revealed that the crack propagation was still unstable for the selected *a*_0_/*L* ratio. This is explained by the fact that similar to the Mode I tests, where specimens were not precracked, the crack propagates from a resin-rich area near the release film crack starter, which is not as sharp as the crack tip resulting from crack propagation; therefore, the load dropped suddenly after crack initiation, thus invalidating the determination of the Mode II crack resistance curves for B and CNT preliminary samples. For this reason, all of the samples were precracked ∼2 mm in Mode I using a wedge before Mode II testing. The tests performed on the baseline samples all resulted in stable crack propagation, as shown for a representative sample in Figure 4a. However, even though the same precracking methodology was used, crack propagation for CNT-reinforced samples was stable only for the first few millimeters of crack propagation and became unstable for crack lengths around 5 mm, the point after which no more data could be collected. This suggests that crack deflection from the reinforced region to the intralaminar region also occurs for Mode II tests. In fact, this crack deviation to the less resistant intralaminar region effectively results in a reduction in crack resistance (a drop in the -curve), violating one of the conditions for crack stability for that sample geometry. Analyzing the crack surfaces, similar features as observed in Mode I are observed in Figure 4c: in the first few millimeters, near the release film, and in the precracking region, the crack propagates somewhat in the CNT-rich interface, but as the crack position progresses, the crack deviates to the intralaminar region. As shown in Figure 4b, where the average -curves for both configurations are presented, the fracture toughness of the CNT-reinforced interfaces tested after initial precracking is around 62% higher than that of the baseline.

### 3.3 Mixed-Mode Test Results

**Figure 5:**
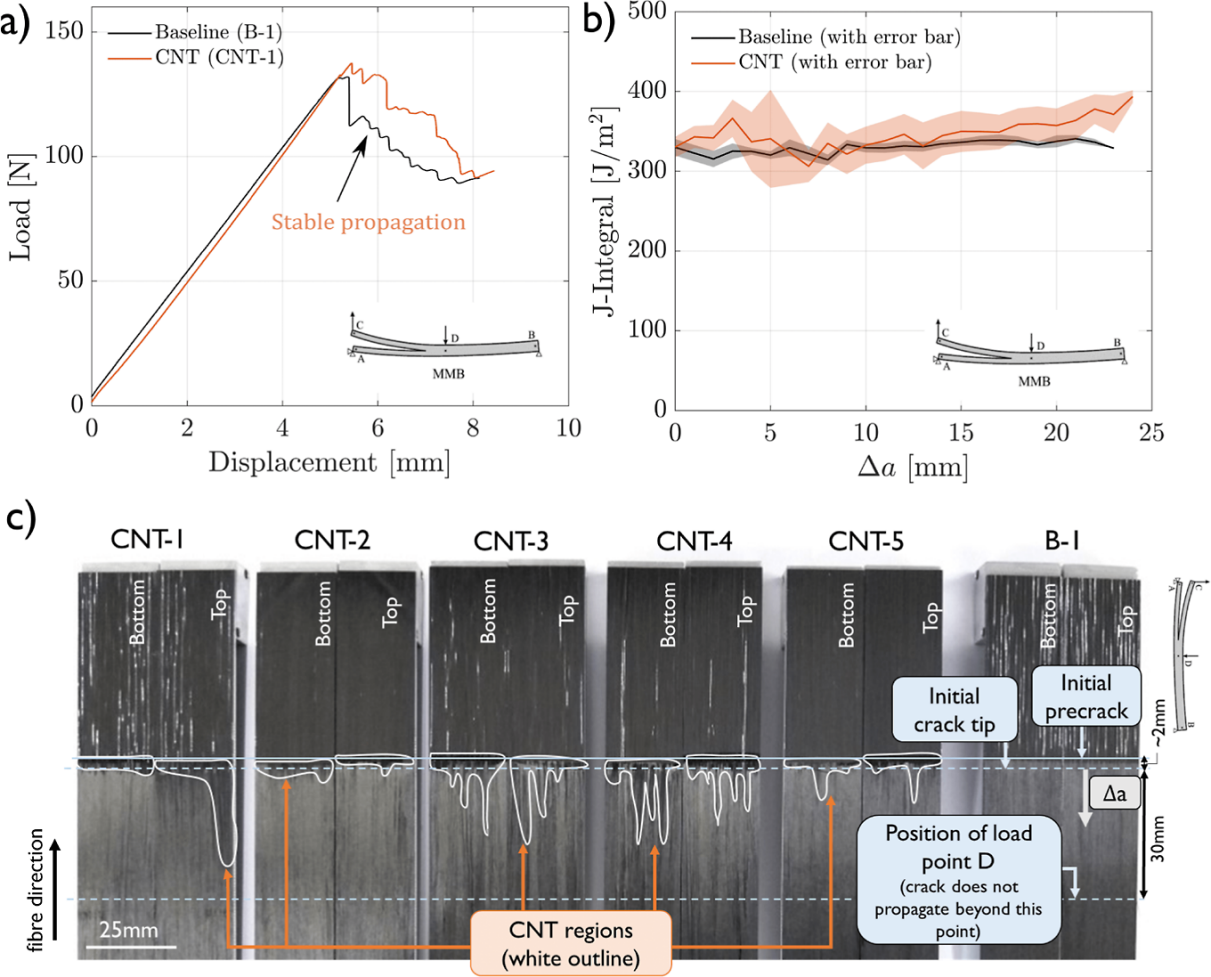
Mixed-mode test results: (a) representative mixed-mode load–displacement curves, (b) average crack resistance curves of baseline (B) and CNT-reinforced (CNT) samples determined following eq 3, and (c) top and bottom crack surfaces of one baseline sample (B-1) and all CNT samples. The CNT-rich crack surfaces are highlighted with a white contour.

Similar to the Mode II samples, from preliminary tests, it was concluded that crack propagation was unstable if the samples were not precracked, and therefore, all of the samples were also precracked ∼2 mm in Mode I using a wedge. Representative load–displacement curves are shown in Figure 5a. From the load and rotation angles at the load introduction points obtained for each specimen, the mixed-mode J-integral was determined following eq 3 and plotted as a function of the crack length. The average -curves for both configurations are shown in Figure 5b. The transition of the crack from the initiation point (at the Teflon insert) to the intralaminar region during precracking in certain samples may be linked to the absence of a toughness increase in mixed-mode conditions when averaged. As in the Mode I and II tests, this transition region is of interest to study in more detail in future work.

## 4 Discussion

For all of the test configurations, the inclusion of VA-CNTs provided additional resistance to crack onset and propagation, resulting in sustained crack deflection from the reinforced interlaminar region into the intralaminar region of the plies; i.e., crack diversion or bifurcation away from the CNT-reinforced interlaminar region was observed within the first 5 mm of crack propagation (extending to 20–25 mm in some cases in smaller strips aligned with the fiber direction). The values of the fracture toughness were calculated for Δ*a* = 0 mm at the point of maximum load for the initiation fracture toughness. For Δ*a* > 0 mm, but (i) before the full transition to the intralaminar region, the fracture toughness values measured and presented in the crack resistance curves (Figures 2b, 4b, and 5b) are not directly related to the CNT-reinforced interface but to a combination of reinforced interlaminar and unreinforced intralaminar regions, and (ii) after the full transition, they are related to an intralaminar region, both with additional crack tortuosity and microfiber nesting effects and therefore will not be commented on in terms of improvements.

**Figure 6:**
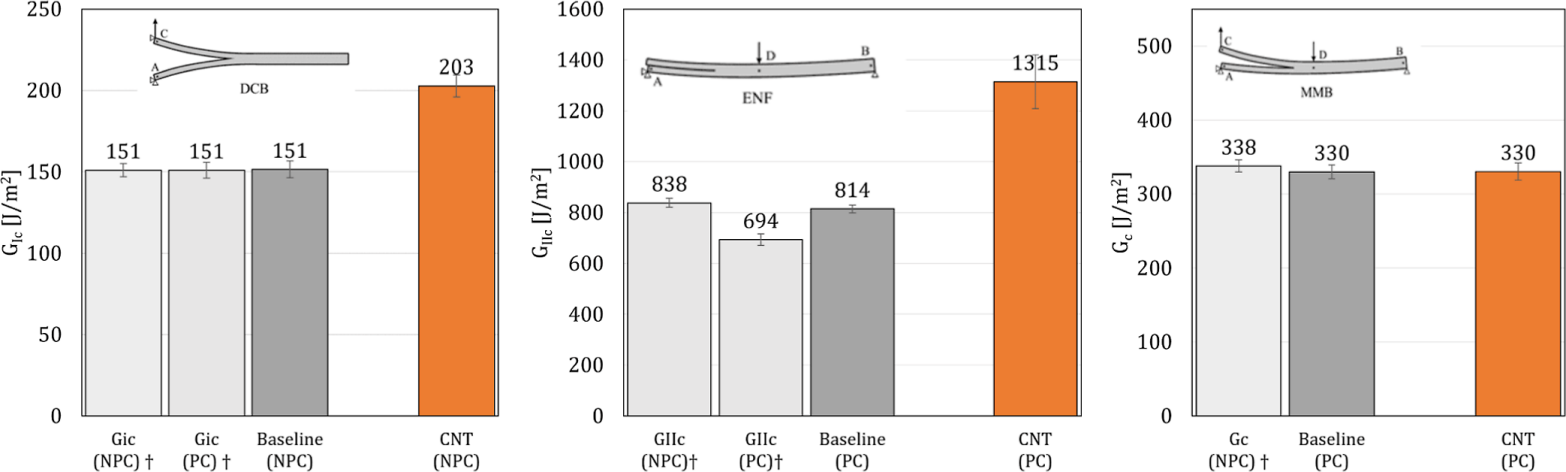
Mode I, Mode II, and mixed-mode initiation fracture toughnesses. ^†^ Values provided by the NIAR obtained for nonreinforced material following the ASTM standards.^30−32^ “NPC” and “PC” stand for “Non-Precracked” and “Precracked”, respectively, as described in ref (31).

The values of the fracture toughness per test and configuration are reported in Figure 6 along with data provided by the National Institute for Aviation Research (NIAR) for the same (nonreinforced) material system following the ASTM standards^30−32^ for comparison. As shown in Figure 6, the data provided by NIAR agree with the values of the fracture toughness obtained for the baseline samples in this work, providing additional confidence that, as expected, the J-integral methods provide accurate values of the fracture toughness for carbon-epoxy composite interfaces at room temperature. Additionally, a direct comparison between the crack resistance curves obtained using the LEFM-based^30,32^ and J-integral-based^33,34^ experimental data reduction methods is presented in Supporting Information C. For nanostitched interfaces, the variability of the two data-reduction methods is subtle and likely more linked to the difficulty in identifying the crack position rather than a larger fracture process zone resulting from the inclusion of CNTs. Given the higher reliability, easier postprocessing, and wider range of validity, more widespread use of J-integral-based data reduction methods can benefit the research and industrial communities. Indeed, in terms of a wider range of validity, only the J-integral reduction allows the quantification of toughness in the transition regions (crack partially in the interlaminar region and partially in the intralaminar region) and in steady state for the cracks that propagate in the intralaminar region parallel to the interlaminar plane as “Mode I/II/mixed intralaminar cracks”. Conversely, the ASTM standard requires the crack to stay at the midplane, which is not found to be the case for the extreme toughening of the CNTs.

As shown in Figure 6, 34 and 62% improvement in the interlaminar Mode I and Mode II initiation fracture toughnesses, respectively, are found due to nanostitching when compared to those of baseline unreinforced interfaces, while no statistically significant improvement was found for mixed-mode fracture toughness. In fact, even though some samples provided improvements up to 30% over the baseline, other MMB samples showed no improvement, likely due to early crack bifurcation. The difference in improvements between Mode I and Mode II toughness is likely attributed to the distinct loading conditions experienced by the CNTs during loading: Mode I testing is anticipated to promote CNT pull-out and sword-in-sheath, whereas Mode II testing may promote more CNT breakage. However, due to in situ observation limitations at the scale at which the mechanisms occur, this remains a speculative hypothesis and should be investigated more fully in future work. The improvements found are well in agreement with the values estimated by Kalfon-Cohen et al.,^28^ where a conservative 15% increase of the Mode I and Mode II fracture toughnesses of the nanoreinforced interfaces was numerically estimated based on finite element analysis of the failure of SBS samples. Previous work^6−8,28,29^ had shown that this type of composite reinforcement based on the inclusion of VA-CNTs in the composite interfaces led to significant improvements in the mechanical performance of composite subcomponents whose failure was dominated by delamination, and this work provides new insights into the mechanics of this technology: delamination onset is, in fact, effectively delayed to higher loads and results in sustained crack deflection into the intralaminar region, ostensibly leading to higher composite strengths. The effectiveness of this nanoreinforcement technique (e.g., improvements in composite strength due to nanostitching) might be more pronounced in thin-ply laminates, as shown by Kalfon-Cohen et al.,^28^ as they have been shown to have a higher resistance to intralaminar crack propagation.^57−69^

## 5 Conclusions

The Mode I, Mode II, and mixed-mode interlaminar fracture toughnesses of thin-ply carbon fiber-epoxy interfaces reinforced by 20 μm tall VA-CNTs were determined following the J-integral-based experimental data reduction methods. As opposed to standard methods for determining the interlaminar fracture toughness, the J-integral-based data reduction methods used in this work do not rely on the subjective measurement of the crack length (although they are plotted against crack length as is usual for -curves), providing more effective data postprocessing and improved reliability of the analysis.

The inclusion of vertically aligned CNTs in the ply interface was shown to provide additional fracture resistance, resulting in sustained crack deflection from the interlaminar region to the intralaminar region of the plies as the crack length increases. Improvements of 34 and 62% on the Mode I and Mode II initiation fracture toughnesses, respectively, are observed; however, due to the advantageous crack deflection phenomenon, the J-integral approach only allowed the evaluation of the fracture toughness of the nanoreinforced interface for Δ*a* = 0 mm as the cracks only propagate through the CNT-reinforced region for very small crack lengths before deviating to within the plies.

This type of interlaminar nanoreinforcement effectively drives crack propagation from the interface to within the ply, resulting in composite structures with improved damage tolerance and fatigue resistance, particularly if used as a selective reinforcement in areas with stress concentrations such as open-holes, L-shapes, bolted joints, or components with ply drops where resistance to delamination onset is critical and fiber bridging may be unlikely given the laminate layup or ply uniformity. The findings presented in this work extend to industries that rely on carbon-fiber advanced composites, including mass-specific applications such as aerospace, wind, automotive, and some infrastructure applications, where lightweight, durable materials are critical for improving structural efficiency. Further studies include CNT reinforcement optimization strategies including densely packed CNTs^70,71^ and scalable manufacturing processes.

## Supporting Information Available

Test setup of the Mode I, Mode II, and mixed-mode tests; CNT-transfer process; and comparison between ASTM vs J-integral calculations (PDF)

## Footnotes

a The stability ratio of *a*_0_/*L* = 0.7 is calculated theoretically from LEFM equations for a sharp crack, assuming a flat -curve (i.e., constant toughness) by imposing  = 0. This is a conservative worst-case-scenario stability limit, verified for a perfectly brittle material: a nonflat rising -curve, i.e., any material with a fracture process zone development or other nonlinearities, should be stable for this geometric ratio if the crack is sharp.
