# Supplementary material for: J-Integral Experimental Reduction Reveals Fracture Toughness Improvements in Thin-Ply Carbon Fiber Laminates with Aligned Carbon Nanotube Interlaminar Reinforcement
Source: ACS Appl Mater Interfaces. 2024 Apr 16;16(16):20980–9. doi: 10.1021/acsami.3c17333 (PMC11056929; doi:10.1021/acsami.3c17333)
Supplement: Supplementary file 1 — am3c17333_si_001.pdf [file am3c17333_si_001.pdf]

Supporting Information: J-integral Experimental Reduction Reveals Fracture Toughness Improvements in Thin-ply Carbon Fiber Laminates with Aligned Carbon Nanotube Interlaminar Reinforcement

Carolina Furtado,[*,†,‡,¶] Reed Kopp,[¶] Xinchen Ni,[§] Carlos Sarrado,[‖,⊥] Estelle Kalfon-Cohen,[¶] Brian L. Wardle,[¶,§] and Pedro P. Camanho[†,‡]

†*DEMec, Faculdade de Engenharia, Universidade do Porto, Rua Dr. Roberto Frias, s/n, 4200-465 Porto, Portugal*

‡*INEGI, Instituto de Ciência e Inovação em Engenharia Mecânica e Engenharia Industrial, Rua Dr. Roberto Frias, 400, 4200-465 Porto, Portugal*

¶*Department of Aeronautics and Astronautics, Massachusetts Institute of Technology, 77 Massachusetts Avenue, Cambridge, MA 02139, United States*

§*Department of Mechanical Engineering, Massachusetts Institute of Technology, 77 Massachusetts Avenue, Cambridge, MA 02139, United States*

‖*AMADE, Polytechnic School, Universitat de Girona, Campus Montilivi s/n, 17073 Girona, Spain*

⊥*AMTEC Composites, C/ Pic de Peguera 15, 17003 Girona, Spain*

E-mail: cfurtado@fe.up.pt

Phone: +351 22 508 1400

A Experimental test setup

A schematic representation of the experimental setup and extracted information used in this work is shown in Figure S.1. The setup is illustrated for a double cantilever sample. A representation of the test setup of the DCB, ENF and MMB specimens is shown in Fig. S.2 along with the sample nominal dimensions.

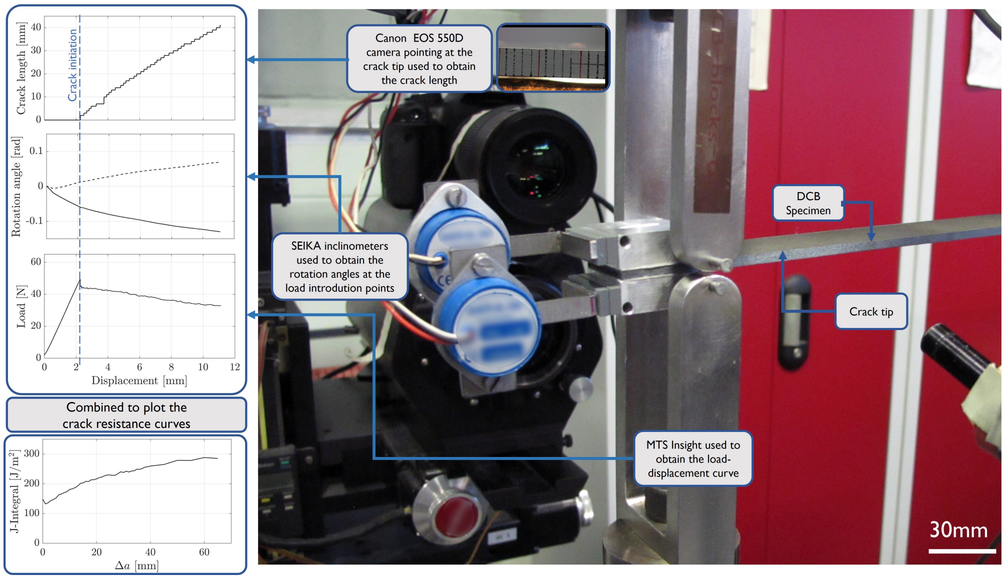

Figure S.1: Schematic representation of the experimental setup and information obtained from the tests (illustrated with a DCB specimen).

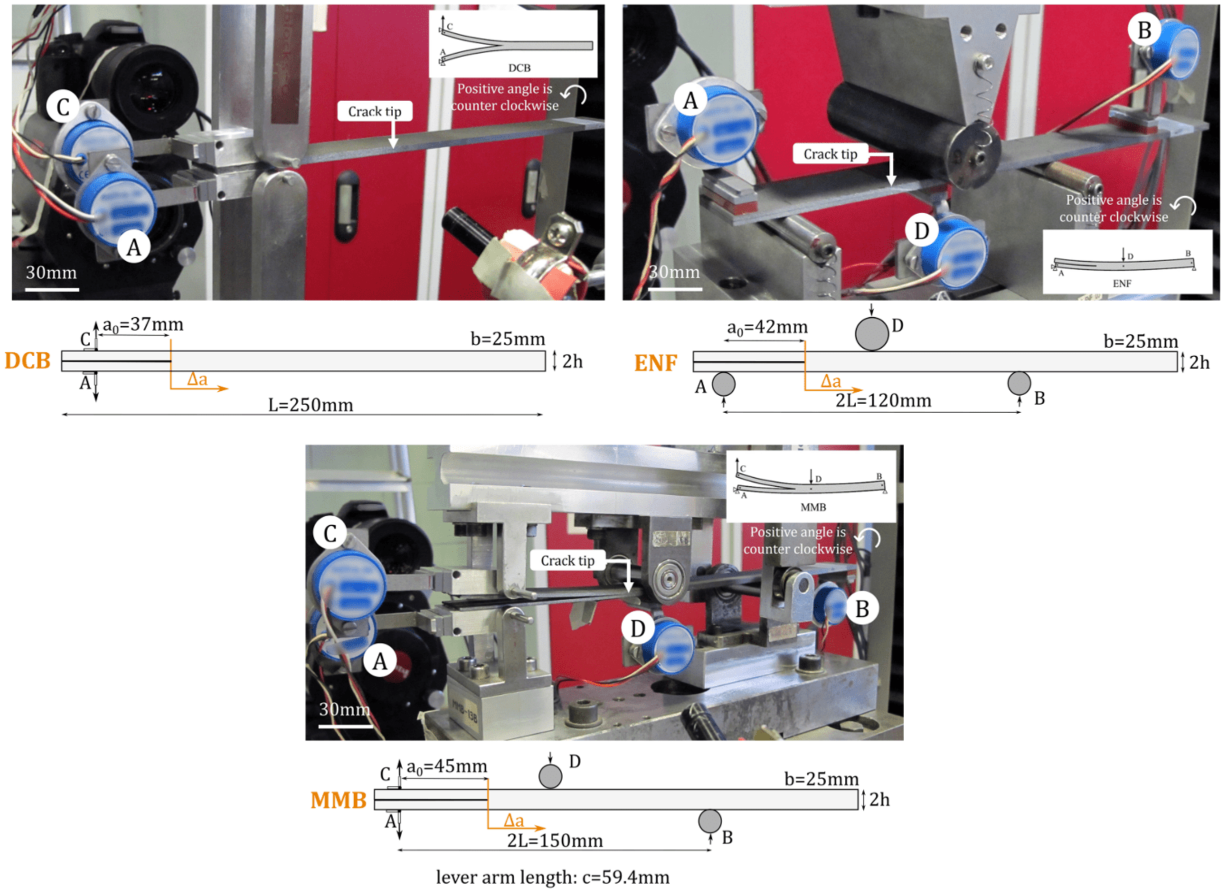

Figure S.2: Experimental setup and nominal dimensions of the Mode I double cantilever beam (left), Mode II end notched flexure test (right), and mixed-mode bending tests (center). The rotation angles at the load introduction points (A, B, C, D) are measured using inclinometers.

B CNT transfer to the composite prepreg

Figure S.3 illustrates the CNT transfer process to the composite prepreg: first, (a) the silicon wafer with VA-CNTs is inverted onto the prepreg, (b) pressure is applied to the backside of the wafer and finally (c) the wafer is removed, leaving the CNTs in the prepreg. (d) The layup process is then continued, resulting in CNT-rich ply-ply interfaces.

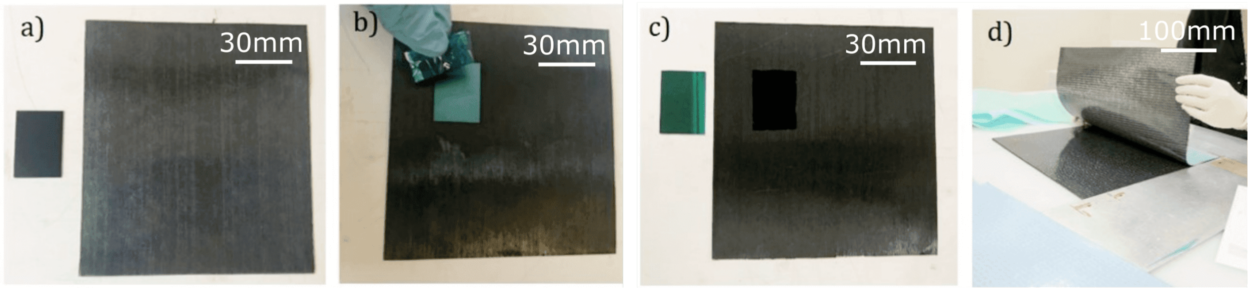

Figure S.3: CNT transfer process: a) the silicon wafer with grown VA-CNTs is inverted onto the prepreg, b) pressure is applied to the wafer so the VA-CNTs are fully transferred onto the prepreg and c) the wafer is removed and d) a new ply is layed-up. Note that the transfer process is demonstrated for a 30 mm x 40 mm CNT forest, however, in this work, for the fracture testing 30 mm x 120 mm forests were used.

C Comparison between LEFM-based and J-integral-based experimental data reduction methods

In this section, a clarification regarding the use of the J-integral approaches to determine the crack resistance curves and how the results compare to the data reduction methods proposed by the ASTM Standards are presented. As mentioned in the main document, the ASTM Standards[1–3] have been largely applied to fiber reinforced polymers composites to determine the fracture toughness and crack resistance curves, however, i) their determination relies on the accurate measurement of the crack length, which can be particularly challenging for Mode II and mixed-mode tests and ii) these methods are based on Linear Elastic Fracture Mechanics (LEFM), i.e. consider that the non-linear deformation at the crack front, and the bridging mechanisms that may be present along the wake of the crack, have a negligible effect. The J-integral closed-form solutions used in this work rely on the determination of the rotation angle of the load introduction points, instead of the crack length. This increases slightly the complexity of the instrumentation, however, it reduces the uncertainty of the results related to the determination of that crack length and greatly simplifies the data post processing. LEFM-based and J-integral-based experimental data reduction methods are

expected to provide the same results in cases of small-scale fracture processes and their results are expected to differ for larger fracture process zone (FPZ) and other toughening effects.[4] Notably, the J-integral approaches can also accommodate a crack plane, as observed here for nanostitching, that is not at the centerline of the laminate (in the interlaminar region) as assumed by the ASTM Standards, and thus only the J-integral approaches properly capture the toughening in the crack transition and propagation regions when the crack bifurcates to an "intralaminar Mode I/II/mixed crack".

In this section, the results obtained using the two types of reduction methods are presented. For the sake of conciseness, the equations proposed by the ASTM standards are only presented for the Mode I tests.[1] For Mode I, there are three data reduction methods for calculating $\mathcal{G}_{Ic}$ given in Ref.:[1] the modified beam theory (MBT), a compliance calibration method (CC) and a modified compliance calibration method (MCC). The values determined by the three different data reduction methods differ by no more than 3.1%.[1] Since it yields more conservative values, the MBT method was used here.

Following the ASTM D5528 standard,[1] the beam theory expression for the strain energy release rate of a perfectly built-in, i.e. clamped at the delamination front, double cantilever beam is given by:

$$\mathcal{G}_{Ic} = \frac{3P\delta}{2ba} \tag{1}$$

where P is the load, δ is the displacement, a is the crack length and b is the specimen's width. However, in practice, this expression will always overestimate the value of $\mathcal{G}_{Ic}$ because the beam is not perfectly built-in (that is, rotation may occur at the delamination front). The modified beam theory (MBT) method proposed by the ASTM standard[1] corrects for this rotation by assuming a slightly larger crack length than the one observed. This correction factor $|\Delta|$ can be obtained experimentally by determining the intersection with the x axis of the least squares plot of the cube root of the compliance, $C^{\frac{1}{3}}$, as a function of the crack length, a, where the compliance is:

$$C = \frac{\delta}{P} \tag{2}$$

The corrected Mode I fracture toughness is then calculated as:

$$\mathcal{G}_{Ic} = \frac{3P\delta}{2b(a + |\Delta|)} \tag{3}$$

As presented in the main document, the J-integral for Mode I proposed by Paris and Paris[5] reads:

$$J_I = \frac{P}{b}(\theta_A - \theta_C) \tag{4}$$

where P is the applied load, b is the width of the specimen and θ_A and θ_C are the angles at the load introduction points.

Figure S.4 shows the Mode I crack resistance curves determined following the ASTM standard (MBT data reduction method) and using the J-integral approach. As shown in Fig. S.4a, for non-reinforced interfaces, there is an excellent agreement between both methods during both initiation and propagation ($\sim$1%).

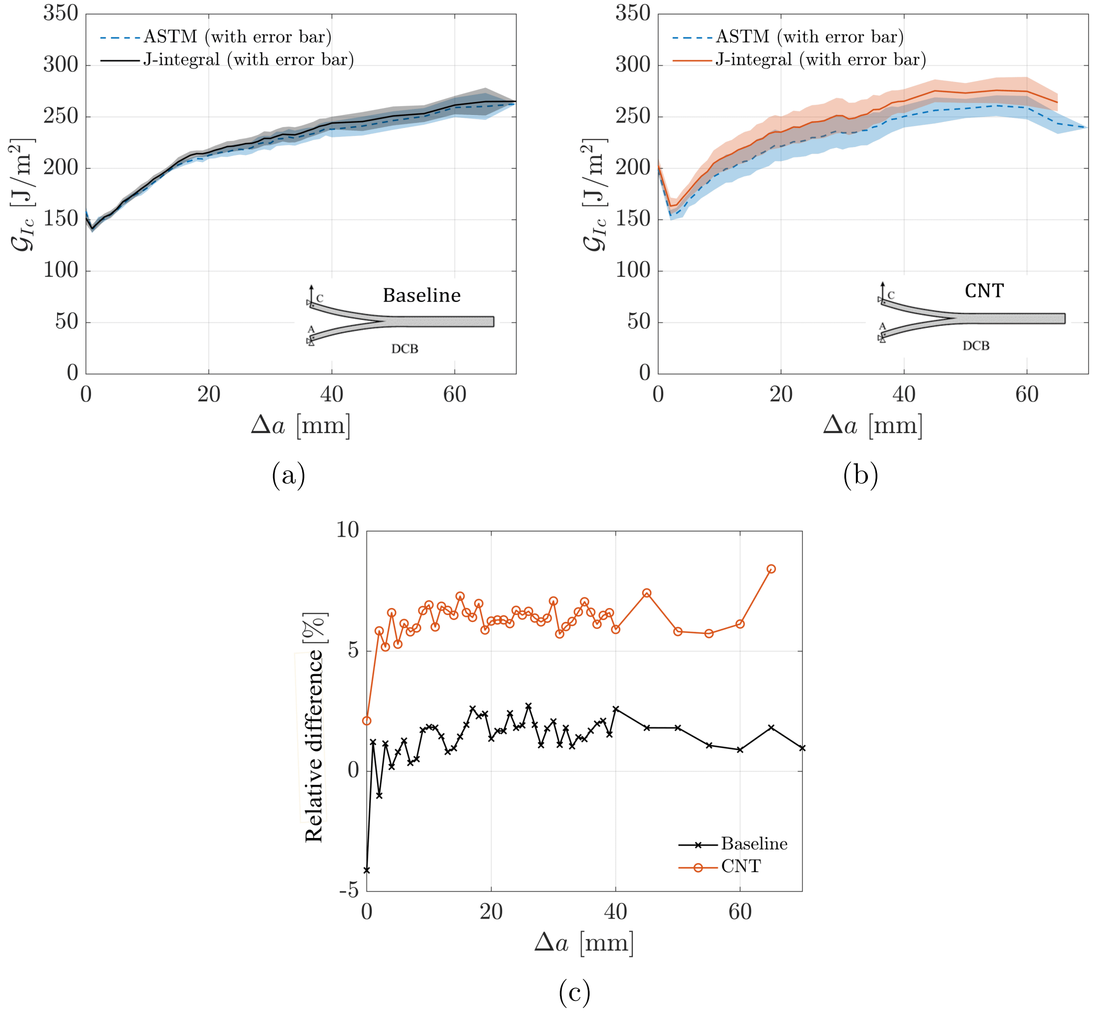

Figure S.4: Mode I crack resistance curves determined following the ASTM standard and using the J-integral approach (a for Baseline samples and b for CNT samples) and relative difference between the J-integral vs. the ASTM Standard (c).

For the CNT-reinforced interfaces (Fig. S.4b), the J-integral is in good agreement for the initiation toughness but diverges 2% to 8% (with a mean of 6.3%) as the crack propagates. This suggests that the correction method provided by the ASTM standard is not able to account for the additional energy dissipation mechanisms or the crack transition and propagation (inside the ply) resulting from the inclusion of VA-CNTs in the interface. Moreover, the ASTM standard methods, contrary to the J-integral solutions, rely on sample symmetry along the crack plane, which is lost as the crack transitions to the intralaminar region. The ASTM standards are, therefore, inappropriate to characterize crack propagation of highly

engineered interfaces as the ones analyses in this work.

Regarding the mixed-mode crack resistance curves, a similar trend is observed: both methods yield equivalent curves for unreinforced baseline interfaces (Fig. S.5b) and for the initiation values of the reinforced samples, but the use of LEFM leads to the over prediction of the propagation fracture toughness of the nano-reinforced samples (Fig. S.5b). Is this case, two factors could be at play for the overestimation of the fracture toughness of the CNT samples using LEFM: i) the under assessment of the position of the crack tip and, ii) the loss of sample symmetry, which invalidates the ASTM method.

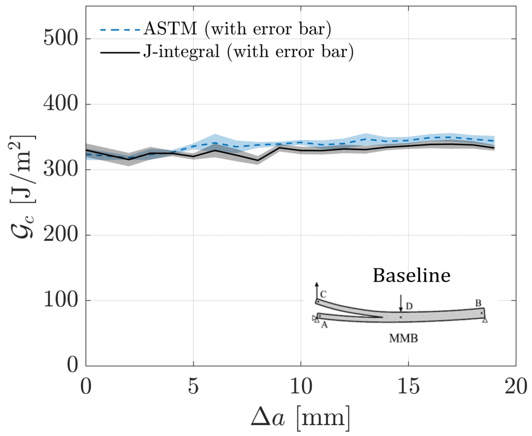

(a)

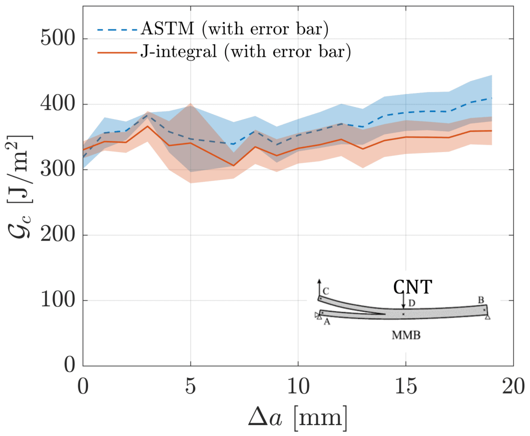

(b)

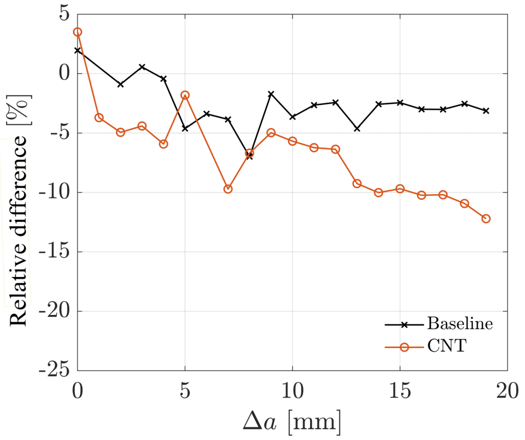

(c)

Figure S.5: Mixed-mode crack resistance curves determined following the ASTM standard and using the J-integral approach (a for Baseline samples and b for CNT samples) and relative errors between the two approaches (c).

The same comparison for the Mode II crack resistance curves was not performed because the ASTM standard only foresees the determination of a single value of the Mode II fracture toughness instead of the crack resistance curves and requires the determination of the compliance calibration coefficients (from tests with different initial precracks) which were not determined in this work.

The LEFM-based and J-integral-based methods presented here are based on distinct assumptions and therefore can provide different results depending on the type of interfaces analysed, particularly for materials that yield large FPZ, such as adhesives joints.[6] For nanostitched interfaces, the methods agree of the initiation values, but diverge as crack propagates. The variability of the two data-reduction methods is likely linked to the difficulty in identifying the crack position and to the loss of sample symmetry, on which the ASTM standards are based on, rather than a larger FPZ resulting from the inclusion on CNTs. Given the higher reliability, easier post-processing and wider range of validity, including transition areas and not well-defined crack tips, it is the authors' conviction that a more widespread use of J-integral-based data reduction methods would benefit the research and industrial community.

References

(1) ASTM D5528 - 13; ASTM D 5528 *Standard Test Method for Mode I Interlaminar Fracture Toughness of Unidirectional Fiber-Reinforced Polymer Matrix Composites*; ASTM International: West Conshohocken, PA.

(2) ASTM D7905 D7905M *Standard Test Method for Determination of the Mode II Interlaminar Fracture Toughness of Unidirectional Fiber-Reinforced Polymer Matrix Composites*; ASTM International: West Conshohocken, PA.

(3) ASTM D 6671 D 6671M *Standard Test Method for Mixed Mode I-Mode II Interlaminar Fracture Toughness of Unidirectional Fiber Reinforced Polymer Matrix Composites*;

ASTM International: West Conshohocken, PA.

(4) Suo, Z.; Bao, G.; Fan, B. Delamination R-curve phenomena due to damage. *Journal of the Mechanics and Physics of Solids* **1992**, *40*, 1 – 16.

(5) Anthony, J.; Paris, P. C. Instantaneous Evaluation of J and C. *International Journal of Fracture* **1988**, *38*, R19–R21.

(6) Sarrado, C.; Turon, A.; Costa, J.; Renart, J. On the Validity of Linear Elastic Fracture Mechanics Methods to Measure the Fracture Toughness of Adhesive Joints. *International Journal of Solids and Structures* **2016**, *81*, 110–116.
